# Computational limitations and future needs to unravel the full potential of 2’-O-methylation and C/D box snoRNAs

**DOI:** 10.1080/15476286.2025.2506712

**Published:** 2025-05-16

**Authors:** Christian Ramirez, Elena Perenthaler, Fabio Lauria, Toma Tebaldi, Gabriella Viero

**Affiliations:** a Department of Cellular, Computational and Integrative Biology (CIBIO), University of Trento, Trento, Italy; b Institute of Biophysics CNR Unit at Trento, Trento Italy; c Department of Internal Medicine, Yale Comprehensive Cancer Center, Yale University School of Medicine, New Haven, CT, USA

**Keywords:** C/D snoRNA, 2’-O-methylation, Nm, ribosome, translation, RNA, cancer, computational biology, epitranscriptome

## Abstract

This review evaluates the current state of C/D snoRNA databases and prediction tools in relation to 2’-O-methylation (2'-O-Me). It highlights the limitations of existing resources in accurately annotating and predicting guide snoRNAs, particularly for newly identified 2’-O-Me sites. We emphasize the need for advanced computational approaches specifically tailored to 2’-O-Me to enable the discovery and functional analysis of snoRNAs. Given the growing importance of 2’-O-Me in areas such as cancer epitranscriptomics, ribosome biogenesis, and heterogeneity, existing tools remain inadequate. As 2’-O-Me gains recognition as a potential biomarker and therapeutic target, more sophisticated methods are urgently needed to improve snoRNA annotation and prediction, facilitating biomedical advancements.

## 1. The coming of age for 2’-O-methylations and C/D box snoRNAs

Among post-transcriptional modifications of ribosomal RNA (rRNA), 2’-O-Methylation (2’-O-Me, also known as Nm) is the most abundant [1,2]. Unlike base modification, 2’-O-Me occurs on ribose, enhancing the stability of the modified nucleotide and protecting it from hydrolytic cleavage [3,4], influencing RNA helix flexibility and interactions within rRNAs and with transfer RNA (tRNA) [5]. Interestingly, variability in 2’-O-Me levels induces alterations in ribosome conformation and selective translation of specific mRNAs [6,7]. Additionally, distinct methylation patterns have been observed during development, suggesting a regulatory role for 2’-O-Me in cellular processes [8–10]. Whilst predominantly identified on rRNAs, 2’-O-Me have been recently observed in other RNA species than rRNA, including snRNAs [11,12], tRNAs [13], and, notably, mRNAs [14,15] as extensively reviewed [16,17].

Over the past decade, advances in epitranscriptomic high-throughput methodologies enabled the fine-mapping of RNA modifications on a genome-wide level. RiboMethSeq, initially proposed by Birkedal and colleagues [18], is a sequencing-based approach that exploits the protection that the methyl group gives to the phosphodiester bond, resulting in a coverage profile with reduced read ends at methylated positions. Alternative sequencing-based approaches exploiting various 2’-O-Me chemical properties, such as 2’-OMe-Seq [19], RibOxi-seq [20], Nm-seq [21], and RimSeq [22], have also been developed. In addition to these methods, the advent of Nanopore sequencing has introduced the possibility of directly sequencing full-length RNA molecules while preserving their native modifications [23]. Although suffering from lower accuracy, this approach has led to the development of the first base-calling algorithms designed to map 2’-O-Me modifications in RNA sequences [14,24–27].

**Figure 1.**
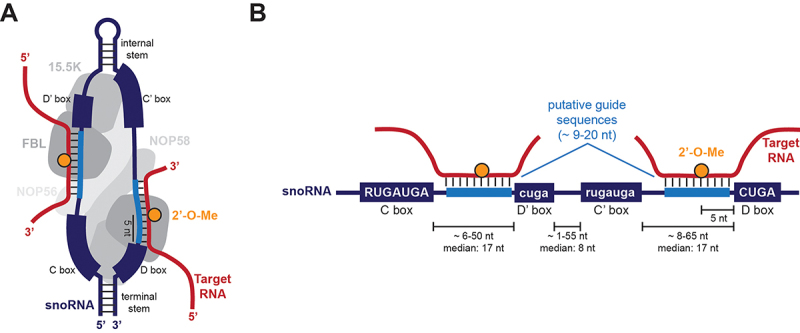
Schematic overview of the human C/D box snoRNP complex (A) and the C/D box snoRNA features (B). The consensus sequence is reported in uppercase for the more conserved C and D boxes and in lowercase for the less conserved C’ and D’ boxes. Distance ranges between boxes as observed in known human snoRNAs [64]. When the distance between the C-D’ or C’-D boxes is lower than 9 nt, the snoRNA lacks the guide sequence.

2’-O-Me is catalysed by distinct methyltransferases, which are specific to different RNA substrates, as summarized in Table 1. In this review, we will focus on the predominant mechanism for site-specific 2’-O-Me deposition in rRNA, which relies on the C/D box small ribonucleoprotein (snoRNP) complex [28]. This complex is composed of a core of proteins, including the methyltransferase fibrillarin (FBL), and of a C/D box small nucleolar RNA (Figure 1(A)) [5,28,29]. C/D box snoRNAs are short RNA sequences, typically 70 to 120 nucleotides in length, which are processed from lariat introns of host genes upon mRNA splicing [30,31]. C/D box snoRNAs are defined by two sets of conserved motifs, the C and the D boxes (with consensus RUGAUGA and CUGA, respectively), and the C’ and the D’ boxes, which usually have less conserved sequences (Figure 1(B)) [32,33]. Between C and D’ boxes or C’ and D boxes, snoRNAs generally contain a guide sequence (also known as antisense element, or ASE), a 9–20 nucleotide long region with reverse complementarity to the specific methylation site in the target RNA [34] . The nucleotide in the snoRNA that corresponds to the methylated nucleotide in the RNA, is located 5 nucleotides upstream of either the D or the D’ box (Figure 1(B)) [30,35,36]. In terms of secondary structure, snoRNAs exhibit distinct folding patterns: nucleotides upstream of the C box and downstream of the D box can form a terminal stem of 4–5 base pairs, whilst nucleotides downstream of the D’ box and upstream of the C’ box can pair to form an internal stem-loop (Figure 1(B)) [37,38]. Beyond their classical role in guiding 2'-O-Me as part of the snoRNP complex, emerging evidence reveals non-canonical functions of snoRNAs [39], including alternative splicing [40,41] and 3’ end processing of mRNA [42]. SnoRNAs are primarily located in the nucleus but can also accumulate in the cytoplasm under stress conditions, suggesting potential roles beyond those identified in the nucleus [43]. Processing of snoRNAs into smaller fragments, termed snoRNA-derived RNAs (sdRNAs) [41,44] has been linked to the formation of other small regulatory RNAs, such as miRNAs [45] and piRNAs [46,47]. Complementary to this topic, a significant fraction of snoRNAs, also called orphan snoRNAs, do not have known targets and functions [22,48]. As they do not pair with rRNA in the nucleolus, it is reasonable to hypothesize that they may have unknown subcellular localization patterns and functions [13,49]. Unlike snoRNAs, which localize to the nucleolus, small Cajal body-specific RNAs (scaRNAs) accumulate within the Cajal bodies – conserved subnuclear organelles in the nucleoplasm – and can guide both methylation and pseudouridylation of small nuclear RNAs (snRNAs), as seen with U85 [50–53]. While scaRNAs are primarily associated with Cajal bodies, it has been suggested that they may also localize in the cytoplasm, challenging the view that they are exclusive to these structures [54].

**Table 1.** List of methyltransferases responsible for the catalysis of 2'-O-Me and of the distinct RNA target substrates.

Methyltransferase	snoRNA-mediated	RNA targets	References
FBL	YES	rRNA, mRNA, snRNA	Omer et al. [55]; Erales et al., [56]; Elliott et al., [57]; Tang et al., [15]
CMTR1 & CMTR2	NO	mRNA 5’ cap	Bélanger et al. [58]; Werner et al., [59]
HENMT1	NO	piRNA 3’ end	Lim et al. [60]
FTSJ1	NO	tRNA	Angelova et al. [61]
FTSJ3	NO	mRNA	Morello et al. [62]; Zhuang et al., [63]

2’-O-Me and C/D box snoRNAs have been implicated as key players in tumorigenesis [65–69]. Notably, mapping of rRNA 2’-O-Me in tumour condition has revealed tumour-specific patterns in lung tumour [24], breast tumour [67] and in leukaemia stem cells [70]. Mutations and expression alterations in snoRNAs have been linked to cancer [71], leading to the definition of oncosnoRNAs [72]. Dysregulation of specific sdRNAs has been martens associated with modulation of gene regulation that favours tumour growth, metastasis and poor clinical outcomes [73,74]. Thus, targeting tumour-specific 2’Ome and snoRNAs presents a promising avenue for both biomarker discovery and therapeutic intervention [72,75,76]. An extensive review on snoRNAs and their link to tumour biology has recently been published by Faucher-Giguère and collaborators [77].

In this context, several computational tools have been developed to identify C/D box guide snoRNAs and assign them to their corresponding RNA modifications. These efforts have culminated in the establishment of dedicated databases that compile both predicted and experimentally validated snoRNAs and snoRNA-guided 2’-O-Me sites.

## 2. Databases of C/D box snoRNAs and 2’-O methylations

Several databases have been developed to catalogue snoRNAs and their associated functions, each offering distinct features and addressing specific needs in the field (Table 2). We will differentiate between databases that contain only information on snoRNAs, or *snoRNA specific*, and the *snoRNA-non specific* databases, whose focus is not snoRNAs, but contain snoRNA-relevant information. Notably, many of these databases also contain information on scaRNAs. However, as this review focuses on C/D box snoRNAs, they will not be discussed in detail.

**Table 2.** List and description of databases providing information on 2’-O-Me and C/D box snoRNAs.

Database	Publication	Link	Last update	Data sources	C/D box snoRNA info	2-O-Me info	Species and C/D box snoRNA/2’-O-Me count
Small Nucleolar RNAs (snoRNAs) from the Yeast Saccharomyces cerevisiae	Piekna-Przybylska et al., RNA, [78]	https://people.biochem.umass.edu/sfournier/fournierlab/snornadb/main.php	2010	GenBank; SGD; CRW2; The European Ribosomal RNA database (discontinued); unpublished data from laboratory; literature	Sequence; genomic locus on SGD (discontinued); predicted and validated target with graphical representation; predicted orthologues	Predicted and validated target 2’-O-Me	S. cerevisiae – 54 snoRNAs; 54 sites
snoRNABase	Lestrade and Weber, Nucleic Acids Res, [79]	https://www-snorna.biotoul.fr/	2007	Literature; Rfam; Plant snoRNA database (discontinued); 5S rRNA Database; RNAdb (discontinued); Small Nucleolar RNAs (snoRNAs) from the Yeast Saccharomyces cerevisiae; Lowe Lab C/D box snoRNA Database; Multalin (phylogenetic alignments of snoRNAs); YASS:: genomic similarity search tool (alignments between human and yeast snoRNAs)	Sequence; host gene; predicted target with graphical representation; predicted orthologues	Predicted target 2’-O-Me	H. sapiens – 269 snoRNAs; 86 sites
Lowe Lab C/D box snoRNA Database	Lowe and Eddy, Science, [80]; Brown et al., RNA, [81]	https://lowelab.ucsc.edu/snoRNAdb/	2016	SnoScan predictions	SnoRNA name; sequence (some); box annotation (some); predicted target	Predicted target 2’-O-Me	S. cerevisiae – 55 snoRNAs A. Thaliana – 139 snoRNAs Archaea – 762 snoRNAs across 14 species
snoPY	Yoshihama et al., BMC Research Notes, [82]	http://snoopy.med.miyazaki-u.ac.jp/snorna_db.cgi	2024	Unclear; possibly snoRNAbase Small Nucleolar RNAs (snoRNAs) from the Yeast Saccharomyces cerevisiae and RNAcentral	Sequence; predicted C/D boxes; genomic locus; host gene; predicted target; predicted orthologues	Predicted target 2’-O-Me	43 species H. sapiens – 418 snoRNAs M. musculus – 327 snoRNAs
snoRNA Atlas	Jorjani et al., Nucleic Acid Res, [22]	http://snoatlas.bioinf.uni-leipzig.de/	2016	HGNC; NCBI; BLAT; sRNA-seq data from ENCODE; snoSeeker (discontinued); snoReport; Sno/scaRNAbase (discontinued); snoRNABase; RNAMDB; The SSU rRNA Modification Database (discontinued); PLEXY; UCSC-Repeat-masker track; snoStrip; In-house Rimseq data; literature	Sequence; predicted C/D boxes; gene locus; conservation; host gene; predicted target	Predicted target 2’-O-Me	H. sapiens – 416 snoRNAs
snoDB 2.0	Bergeron et al., Nucleic Acid Res, [64]	https://bioinfo-scottgroup.med.usherbrooke.ca/snoDB/	2022	snoRNABase; snoPY; snoRNA Atlas; RNAcentral; Ensembl; RefSeq; NCBI; HGNC; Rfam; The Human Protein Atlas; RISE DATABASE; ENCODE; snoRNA abundance datasets; literature	Sequence; predicted C/D boxes; predicted structure; conservation; gene locus; host gene; predicted interactions; expression; snoRNA-protein interactions	Predicted target 2’-O-Me	H. sapiens – 1107 snoRNAs
PISNO	Wang et al., Cancer Lett., [83]	https://hanlaboratory.com/PISNO	2024	miRNAseq data (Gong et al., 2017); VAEN, CanceRxTissue; TIMER; TIMER2.0; xCell; MCP-counter; CIBERSORT; EPIC; quanTIseq; immunedeconv R package; ImmuneCellAI	snoRNA name; genomic locus; association with cancer immunotherapy	None	H. sapiens – 31 cancer types with 386–486 associated snoRNAs
RMBase v3.0	Xuan et al, Nucleic Acid Res, [84]	https://rna.sysu.edu.cn/rmbase3/	2024	Public datasets of Nm-seq; Ribometh-seq; RibOxi-seq and 2OMe-seq; MODOMICS; snoPy; Small Nucleolar RNAs (snoRNAs) from the Yeast Saccharomyces cerevisiae, snoRNAbase; GENCODE; NCBI; Ensembl; snoSeeker (discontinued)	Predicted guide snoRNA	Gene locus; surrounding sequence; predicted secondary structure; cell/tissue; sequencing type; conservation; writer	42 species H. sapiens – 152 snoRNAs; 8490 sites M. musculus – 81 snoRNAs; 1193 sites
Rfam	Ontiveros-Palacios, Nucleic Acids Res., [85]	https://pmc.ncbi.nlm.nih.gov/articles/PMC165453/	2024	Uniprot; Protein Information Resource; R-scape; ViennaRNA; fasttree; literature	Sequence; secondary structure prediction; predicted orthologues; predicted phylogenetic tree	None	26106 species H. sapiens – 2342 snoRNAs M. musculus – 1086 snoRNAs

### snoRNA specific

Given that the majority of initial research on snoRNAs was conducted in yeast [33], the first dedicated snoRNA database was developed for Saccharomyces cerevisiae, titled *Small Nucleolar RNAs (snoRNAs) from the Yeast Saccharomyces cerevisiae*, from Samarsky and Fournier [86]. This database collects data from published literature, offering a comprehensive repository of yeast snoRNAs. It includes information on their predicted and/or experimentally validated 2’-O-Me target sites, shown through an intuitive graphical interface that visualizes the interactions between snoRNAs and their corresponding targets. Established in 2006 by Lestrade and Weber, *SnoRNABase* [79] expanded the focus to human C/D box snoRNAs and their predicted 2’-O-Me modifications, with interactions presented through a graphical interface and data collected from literature available at the time, including orthology information between human and yeast. The lack of updates since 2007 significantly reduces its utility compared to more modern resources. Similarly, the Lowe Lab *C/D box snoRNA Database* [80,81] collects snoRNA data from yeast, archaea, and *Arabidopsis thaliana*, based on computational predictions via *SnoScan* [80]. However, its reliance on predictions rather than experimental validation limits its reliability. Expanding beyond individual species, the snoRNA Orthological Gene Database (*snOPY*) [82], launched in 2008 by Yoshihama and colleagues, identifies orthologous snoRNAs across 43 species and assigns their target 2’-O-Me sites. Whilst valuable for comparative and evolutionary studies, greater clarity on its data sources would enhance its utility and reproducibility for the scientific community. In 2016, *snoRNA Atlas* [22] provided a major update to human snoRNA research. Taking advantage of high-throughput data produced by the authors throughout the study [22], public databases, ENCODE small RNAseq data [87], and computational tools – *snoSeeker* [88], *Snoreport* [89], *PLEXY* [90], *snoStrip* [91] – *snoRNA Atlas* redefined the human snoRNA – target RNA predicted interaction network. They identified 416 C/D box snoRNAs, providing a comprehensive view of these molecules and their interactions.

Currently, *SnoDB* [64,92], initially developed in 2019 and updated to version 2.0 in 2022, addresses many of the limitations identified in earlier databases. By consolidating information from previous databases [22,79,82,85,87,93], literature [2,67,94–97], and in-house datasets on snoRNA expression levels, it provides extensive details on snoRNA features, tissue-specific expression, predicted and validated interactions, conservation and predicted secondary structure. This makes SnoDB one of the most comprehensive and up-to-date resources for snoRNA research, although limited to human snoRNAs.

Focusing on cancer immunotherapy, the Pharmacogenomic and Immune landscape of snoRNA (*PISNO*) [83], presents a comprehensive investigation of the influence of snoRNAs on drug response and tumour microenvironment. This resource integrates snoRNA expression data derived from miRNAseq experiments across 31 cancer types [71] with predictive models for pharmacogenomic analysis [98,99] and immune cell population estimation [100–108]. PISNO serves as an essential database for researchers aiming to identify snoRNA biomarkers or therapeutic targets, with the potential to drive the development of more effective and personalized anti-cancer treatments.

### snoRNA non-specific

*Rfam* [85], a broader database for studying non-coding RNAs, catalogues RNA families based on conserved sequences and experimentally validated or predicted structures in more than 2600 species. It provides curated alignments and structural models of C/D box snoRNAs, thus enabling functional annotation and comparative analyses. Complementing these resources, *RMBase* [84], originally released in 2016 [109] and updated to *RMBase 3.0* in 2024, focuses specifically on RNA modifications. It aggregates data from public but unspecified datasets of Nm-seq, Ribometh-seq, RibOxi-seq, and 2OMe-seq, along with computational predictions via the now-discontinued snoSeeker [88]. RMBase includes annotations of 2’-O-Me sites and their guide snoRNAs, offering information on modifications in diverse RNA types, including mRNA, and detailing the cell lines, tissues, and writer enzymes associated with these modifications.

### Database conclusions

Notably, *snoRNABase* [79], *snOPY* [82], *snoRNA Atlas* [22], SnoDB 2.0 [64], Rfam [85], and RMBase 3.0 [84] also include information on scaRNAs and their predicted or validated guided sites. Overall, available databases have significantly advanced snoRNA research. Nevertheless, limitations in updates, coverage, and accessibility of some tools underscore the ongoing need for further development in this area. Each resource, with its unique contributions and drawbacks, highlights the importance of refining and expanding databases to keep pace with the growing complexity of snoRNA and 2’-O-Me research.

## 3. Computational methods for C/D box snoRNAs and 2’-O methylation sites prediction

**Figure 2.**
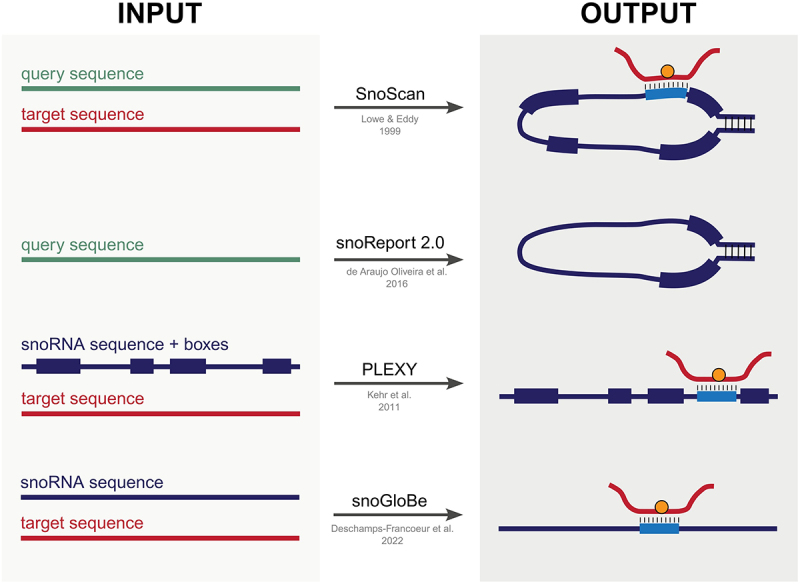
Schematic representation of input requirements and outputs returned by current computational methods for snoRNA predictions.

Due to the distinct characteristics of snoRNA biology, various computational prediction tools have been designed to annotate snoRNAs within genomes and identify the target 2’-O-Me sites they guide (Figure 2 and Table 3). The first tool designed for the prediction of C/D box snoRNAs was *Snoscan* [80], introduced in 1999 by Lowe and Eddy as a command-line program written in C, and later adapted into a web-based tool with graphical interface. Snoscan employs a probabilistic modelling approach trained on a dataset of 35 human snoRNAs and 9 yeast snoRNAs. It takes as input query sequences where the snoRNAs should be located and rRNA sequences that contain the corresponding methylation sites, which can also be specified as input. Utilizing probabilistic modelling, Snoscan identifies snoRNAs based on their characteristic components in the query sequences and predicts their guided methylation sites within the rRNA. Although the core code has not been updated since its release, Snoscan is a widely used resource due to it being user-friendly and efficient. However, it is limited by not being able to easily handle long query sequences, such as human or mouse chromosomes.

**Table 3.** List and description of computational tools for C/D box snoRNA identification and assignment to 2’-O-Me.

Tool	Publication	Link	Last update	Data sources	Input	Output
SnoScan	Lowe and Eddy, Science, [80]	https://lowelab.ucsc.edu/snoscan/	2020	35 human and 9 yeast C/D box snoRNAs	query sequences; RNA sequences; 2-O-Me sites (optional)	snoRNA sequences; features; guide sequence-RNA interaction
SnoReport 2.0	de Araujo Oliveira et al., BMC Bioinformatics, [110]	https://joaovicers.github.io/snoreport2/index.html	2016	2 datasets of C/D box snoRNAs; Vienna RNA package	query sequences	snoRNA sequences; C and D box annotation
PLEXY	Kehr et al., Bioinformatics, [90]	http://legacy.bioinf.uni-leipzig.de/Software/PLEXY/	2011	RNAplex; snoRNA-rRNA interaction rules	snoRNA sequences; RNA sequences; box annotation	snoRNA guide sequence-RNA interactions
snoGloBe	Deschamps-Francoeur et al., Nucleic Acids Res, [111]	https://github.com/scottgroup/snoGloBe	2022	RNA-RNA interactions from: PARIS, LIGR-seq and SPLASH databases, snoRNABase predictions, literature	snoRNA sequences and ids; genome annotation; chromosome sequences	snoRNA-RNA interactions

More recent tools focus on either predicting snoRNA sequences in a genome or predicting snoRNA – RNA interactions. In the first category, *SnoReport 2.0* [110], a Python-based command-line tool originally released as SnoReport in 2008 [89], utilizes Support Vector Machine methods to identify C/D box snoRNAs based on snoRNA features, including C and D box, terminal stem pairing, and predicted secondary structure with RNAfold [112]. SnoReport 2.0 can detect snoRNA sequences starting from genomic query sequences as input. However, it does not assign the identified snoRNAs as guides for a target site. Moreover, SnoReport 2.0 validation based on datasets of experimentally validated snoRNAs reveals moderate accuracy [110], highlighting the need for further refinement. Unfortunately, no updates have been applied since 2016.

Regarding snoRNA – RNA interaction prediction, *PLEXY* [90], a Perl-based command-line program developed in 2011, is a dynamic programming algorithm designed to compute thermodynamically optimal interaction between C/D box snoRNAs and their putative target RNAs. PLEXY requires C/D box snoRNA sequences in FASTA format, with annotated C and D boxes in the headers, and target RNA sequences. By leveraging RNAplex [113] and interaction rules defined by [114], PLEXY computes stable duplexes between snoRNA guide sequences and target rRNA. The found interactions are then filtered and ranked based on their duplex energies. As such, PLEXY is well-suited for identifying target methylation sites target of known snoRNA sequences. Developed by the same research group, snoStrip [91] is a command-line tool that functions as a computational pipeline to annotate snoRNAs in a genome using known snoRNAs from another species. However, it suffers from limited documentation and has seen minimal adoption beyond its original publication, reducing its accessibility for broader applications.

Finally, developed by the same team of snoDB, *snoGloBe* [111] is a Python-based command-line tool that utilizes a gradient boosting classifier from scikit-learn (v0.21.3) to identify binding sites for C/D box snoRNAs. This model is trained on experimentally identified snoRNA-RNA interactions from PARIS [115], LIGR-seq [116], and SPLASH [117] – methods described in detail later. These experimentally validated interactions are complemented by data retrieved from the literature and predicted interactions reported in snoRNABase [79]. SnoGloBe takes as input snoRNA sequences, a text file containing snoRNA IDs, a genome annotation file (in GTF format), and a directory containing individual chromosome sequences. With respect to previous target prediction tools, snoGloBe does not only look for interactions between the guide sequence of the snoRNA and the target RNA, but finds possible interactions located in any part of the snoRNA sequence. Therefore, it can be particularly useful to find snoRNA-RNA interactions not 2’-O-Me related.

It is important to note that current computational tools for RNA prediction are primarily based on standard snoRNAs, and no dedicated tool has yet been developed for predicting scaRNAs.

To complement the main discussion on computational prediction tools, it is relevant to mention sequencing-based methods and ad hoc bioinformatics pipelines which are instrumental in identifying C/D box snoRNA–RNA interactions. Over the past decade, to capture RNA-RNA interactions, several high-throughput approaches have been developed, such as PARIS (Psoralen Analysis of RNA Interactions and Structures) [115], LIGR-seq (Ligation of Interacting RNA followed by high-throughput sequencing) [116], SPLASH (Sequencing of Psoralen-crosslinked, Ligated, And Selected Hybrids) [117], RIC-seq (RNA in situ conformation sequencing) [118], and KARR-seq (kethoxal assisted RNA-RNA interaction sequencing) [119]. These techniques employ crosslinking strategies combined with proximity ligation, sequencing and dedicated bioinformatics pipelines to process chimeric reads and reconstruct interaction networks. SnoRNA–RNA chimeras were investigated in 2017 [120] starting from PAR-CLIP of core snoRNP proteins [121] followed by RiboMeth-seq to map 2’-O-Me sites potentially guided by the detected interactions. More recently, snoRNA-tRNA interaction network was revealed [122] by applying PARIS2, a refined protocol and pipeline for duplex structure predictions [123], while previously unrecognized snoRNA-mRNA duplexes were identified by snoKARR-seq (snoRNA enriched KARR-seq) [124]. These sequencing approaches provide powerful tools for the validation of snoRNA-RNA interactions. However, they lack direct validation of snoRNA-mediated 2’-O-methylation and instead rely on parallel methods like RiboMeth-seq to infer the functional outcomes of these interactions.

Complementary to snoRNA prediction tools, computational approaches for the prediction of 2’-O-Me sites have been developed. These tools include the web servers *i2OM* [125], *H2Opred* [126], *Meta-2OM* [127], and *Nmix* [128], which use machine learning and deep-learning techniques to predict methylation sites starting from human RNA sequences. Additionally, a variety of studies have used machine learning approaches to detect 2’-O-Me sites from Nanopore direct RNA sequencing [14,25–27]. These studies open exciting avenues for the prediction and fine mapping of 2’-O-methylation across diverse tissues and organisms.

The computational tools presented here are highly useful for identifying guide snoRNAs from small RNA-seq data and mapping the 2’-O-Me sites they guide. However, some limitations remain to be addressed. In the ‘Current limitations and future directions’ section, we will explore these challenges and propose potential directions for future research.

## 4. Current limitations and future directions

Available databases and computational tools are great resources for characterizing C/D box snoRNAs and the 2’-O-Me sites they guide. Nonetheless, significant challenges arise when attempting to predict all possible snoRNAs in a large genome, such as *H. sapiens*. While there are available tools capable of performing this task [80,110], they currently present limitations such as prohibitive computational time or lack of recent updates. Furthermore, certain methods exclude key snoRNA features, such as C and D boxes, in their output, which can complicate downstream analyses with tools for the assignment of predicted snoRNAs to specific methylation sites [90].

In keeping with these issues, the requirement of prior annotation of snoRNA features as input for the 2’-O-Me site assignment not only reduces the user-friendliness of tools, but also increases the potential for errors. Furthermore, the general lack of updates results in dependencies on outdated computational frameworks. These are often incompatible with modern environments, which makes them difficult to instal and use effectively. As a result, the currently available tools may not fully meet the needs for genome-wide prediction of novel snoRNAs and the assignment of the corresponding 2’-O-me sites. These challenges are exacerbated when working in the reverse direction. For instance, the identification of guide snoRNAs corresponding to novel 2’-O-me sites detected by a RiboMethSeq experiment is overly complex. The only feasible approach involves predicting all snoRNAs across the genome and attempting to assign them to the 2’Ome sites of interest. This process is computationally intensive and may yield limited success.

A further challenge with the available tools lies in the quality and completeness of data they rely on, particularly for machine learning models that require a robust training set [129]. Most of the human guide snoRNA – 2’-O-Me assignments have been derived primarily through predictive analyses, while limited experimental validation has been performed [7,130–132]. In fact, no comprehensive collection of experimentally validated human snoRNA-guided 2’-O-Me sites currently exists. This reliance on computational predictions introduces potential inaccuracies, since a perfect match between the snoRNA’s guide sequence and the target rRNA is often assumed to be a functional interaction. However, this assumption is not always accurate and should be rigorously tested. A notable example illustrating this comes from the work by Jansson and colleagues [7]. In their study they examined human SNORD45C, predicted by multiple sources to guide methylation at both 18S:159 and 18S:174 due to a perfect sequence match with these rRNA targets. However, through experimental validation they demonstrated that SNORD45C only guides the 18S:174 site. Therefore, experimental validation of snoRNA guided 2’-O-Me, for example through knocking down specific snoRNAs and measuring changes in 2’-O-Me levels, is a crucial step for validating predicted interactions. Without such a validation, the reliability of the data used for the development of prediction tools is compromised, in turn affecting the accuracy of subsequent 2’-O-Me assignments. Similarly, current databases present many instances where multiple snoRNAs are predicted to guide the same 2’-O-Me site. Despite these overlaps being systematically catalogued in databases that allow searches by target sites [64,79,82,84], it is often unclear which snoRNA is the primary guide. A striking example concerns 2’-O-Me site 18S:159, which is predicted by multiple sources to be guided by SNORD45 A/B/C and by 42 copies of SNORD115. Due to the aforementioned issue of limited experimental validation, contributions of different snoRNAs remain undetermined, highlighting the urgent need for systematic experimental studies to refine predictions and improve database accuracy.

An additional annotation issue arises with orphan snoRNAs. In existing databases, snoRNABase [79] explicitly categorizes them as orphans, snOPY [82] searches for snoRNAs with unknown targets, and snoDB [64] enables sorting by ascending target number. Among available computational tools, only Snoreport 2.0 [110] is capable of predicting snoRNA sequences independently of their complementarity with rRNA sequences. Given emerging evidence that orphan snoRNAs may play non‐canonical roles [13,49], functional validation is essential to uncover their potential targets and elucidate their biological functions.

To fully understand snoRNAs’ role, there is an urgent need for comprehensive data collection across multiple information layers, as summarized in Table 4. These layers include sequence, structure, expression, subcellular localization, conservation, variation, functional role, disease associations, and target 2’-O-Me sites.

**Table 4.** The ten information layers necessary to unlock our understanding of snoRNAs. Each layer is associated with current challenges, both experimental and computational.

	Information layer	Challenge (experimental and computational)
1	**coordinates/sequence**	*define exact start/end, distinguish snoRNA (and snoRNA fragments) from pseudogenes*
2	**boxes and guide**	*define guide boundaries, identify real C/D boxes (short degenerate motifs with multiple possible combinations)*
3	**structure**	*obtain experimental structures, predict the structure with associated proteins*
4	**expression**	*structure and length heterogeneity affect sequencing efficiency, develop counting strategies for abundance estimation*
5	**conservation**	*improve multi-species annotation and snoRNA evolutionary models*
6	**localization**	*define nuclear and possible non nuclear localization*
7	**rRNA modifications**	*match orphan snoRNAs and novel 2-O -me*
8	**functional role**	*experimental validation with knock-out, silencing, crispr or similar techniques*
9	**variation**	*identify snoRNA variants*
10	**association to disease**	*define snoRNA biomarkers and develop snoRNA drugs*

In the past decades, ribose 2’-O-Me have been shown to represent the most abundant post-transcriptional modifications of ribosomal RNA [95]. Being predominantly located in critical ribosomal regions [56,133], these modifications are thought to actively participate in translational control by generating heterogeneous ribosome populations with specific structure and functions [7,9,65,67,96]. Since accumulating evidence demonstrates that cancer ribosomes display specific methylation patterns [2,67,134], future research should focus on understanding the regulation and functional consequences of rRNA 2’-O-Me alterations. Thus, there is an urgency to comprehensively identify the snoRNAs guiding 2’-O-Me deposition to fully understand their role in physiological and disease conditions. This will open up new avenues for leveraging 2’-O-Me in the clinic as biomarkers and therapeutic targets.

## Data Availability

No datasets were generated or analysed during the current study.

## Abbreviations

2’-O-Me: 2ʹ-O-methylation
rRNA: ribosomal RNA
tRNA: transfer RNA
snoRNA: small nucleolar RNA
sdRNAs: snoRNA-derived RNAs
